# Supplementary material for: A novel methionine nanoparticle in broiler chickens: Bioavailability and requirements
Source: PLoS One. 2024 Apr 17;19(4):e0302230. doi: 10.1371/journal.pone.0302230 (PMC11023566; doi:10.1371/journal.pone.0302230)
Supplement: Attachment — Submitted filename: Comments to Authors PONE-D-23-43596.doc [file pone.0302230.s002.doc]

**Comments to the Authors**

I have conducted a thorough examination of the manuscript, and it is evident that substantial revisions are needed. Below are some of my comments:

**Comment #1. Line 23, 24.** "p < 0.05" should be italicized. Pleasse check the whole manscript.

**Comment #2. Abstract.** In conclusion, it would be beneficial to include information regarding the optimal dose of methionine supplementation. Please consider adding this detail.

**Comment #3: Keywords.** It would be beneficial to enrich the study by including important keywords related to the research. Consider incorporating specific terms such as the name of the animal involved and the substance used.

**Comment #4. Introduction.** Consider emphasizing the research problem and the novelty more towards the end of the introduction for better clarity. Please rewrite it clearly with mention the specific parameters to achieve the aim.

**Comment #5: Line 145.** Preparation of Methionine Nanoparticles. It would be beneficial to provide references for the methodology used in the preparation of methionine nanoparticles. This helps readers and researchers to access additional information, ensuring transparency and credibility in the study.

**Comment #6: Line 207 and 208.** Inconsistent Figure References. Please use either the abbreviation 'Fig.' or the full name 'Figure' consistently for references such as 'Fig. 1a' and 'Figure 2b' to maintain uniformity throughout the document. Please check the entire manuscript.

**Comment #7: Results.** In the section spanning from Line 206 to 212, it would be beneficial to include a descriptive title for the description of this result. A clear and concise title will provide readers with a preview of the content, aiding in the overall organization and comprehension of the manuscript.

**Comment #8: Results. In Line 213,** the term "Analysis of variance" is used as a title. Please consider if this title accurately reflects the specific content and findings presented in this section. If possible, provide a more specific and descriptive title that aligns with the nature of the results discussed, ensuring clarity for readers.

**Comment #9: Line 247, 255, 262, 269, 276, etc.** Inconsistency in Reference Format. The citation 'Aviagen (2022)' appears multiple times throughout the manuscript. Please clarify whether 'Aviagen (2022)' is a report, a publication, or another type of reference.

**Comment #10: Conclusion.** It is recommended to place the 'Conclusion' section under a separate heading for improved organization and readability. This would enhance the structure of the manuscript and make it easier for readers to navigate and comprehend the key findings and implications.

**Comment #11: Tables.** The manuscript makes references to several tables, but they appear to be missing. Please ensure that the tables mentioned in the text are included in the manuscript.

**Comment #12: Figures.** It is observed that all the figures in the manuscript are without captions. Please check it.

**Comment #13.** I have noticed a few minor errors and spelling mistakes scattered throughout the manuscript. To enhance the linguistic quality of the manuscript, I would recommend considering professional language revision.
