# Supplementary material for: A novel methionine nanoparticle in broiler chickens: Bioavailability and requirements
Source: PLoS One. 2024 Apr 17;19(4):e0302230. doi: 10.1371/journal.pone.0302230 (PMC11023566; doi:10.1371/journal.pone.0302230)
Supplement: Attachment — Submitted filename: PONE-D-23-43596_Review.docx [file pone.0302230.s003.docx]

I am pleased to participate in reviewing this interesting research manuscript. The study reported a patented formulation of DL methionine into nanoparticles and investigated the positive effect of DL methionine supplementation on broiler performance. The study also statistically compared the performance parameters in response to DL methionine forms “powder and nanoparticles” supplementation using well-designed statistical models. However, I believe that the manuscript needs a major revision to reduce the redundancy and fulfill the following comments.

- The standard performance parameters (body weight and FCR) should be mentioned at least to know how far the results are, when compared to the standard.
- I admire your statistical proficiency. Therefore, I would recommend adding a power analysis for the used sample size.
- Figures’ captions should be provided. The caption should include detailed information about the figure that can be stand-alone to understand the figure without reading the manuscript.
- Where are the Tables (1 – 3), I could not find them in the manuscript?!!
- Line (L) 19: “….to create 11 experimental diets including a basal diet and…” it is confusing. Please add a comma “,” before “including”
- L 43: Reference [3] seems irrelevant to the reported information. Please cite a more relevant reference.
- L 145 – 153: What was the source of lipid used in the methionine nanoparticle formation?
- L 234: Remove “FCR BMY”.
- L (322 – 343): “Nanotechnology has been increasingly ……..” Redundant information and unnecessary details should be omitted. It is already explained in the introduction. Please remove the whole paragraph L (322 – 343).
- Five statistical models were used to analyze FCR, TMY, and MDA (Fig 4, 6, and 7), while only three statistical models were used to analyze BWG and BMY (Fig 3 and 5). I believe that you should be consistent. Please explain if you have a rationale for not being consistent.
- Where is the “Conclusion”?! I guess that it is the last paragraph. Either you call the “Discussion” section (Discussion and conclusion), or you add the section title “Conclusion” before the last paragraph.
- Fig. 8 has not been mentioned in the manuscript. Either you discuss the figure or remove it.
- The supplementary data also has not been mentioned in the manuscript. Further, the “CSV” tables contain values with column titles “Met, BRST, and THIGH”. Should the reader guess?!!!
